# Case of a Scirrhous Affection of the Stomach; with an Account of the Appearances on Dissection

**Published:** 1790

**Authors:** Robert Graves

**Affiliations:** Physician at Sherborne in Dorsetshire, and Extra-Licentiate of the College of Physicians, London


### IV. *Cafe of a fcirrhous Affection of the Stomach; with an Account of the Appearances on Diffection. By* Robert Graves, *M. D. Phyfician at Sherborne in Dorfetfhire, and Extra-Licentiate of the College of Phyficians, London.*

ELIZABETH BURROWS, aged twenty-four years, came under my care Auguft 30th, 1788, being admitted an in-patient of the hofpital at Northampton on account of a painful and fevere vomiting, from the long continuance of which fhe was then reduced to a ftate of great weaknefs and emaciation. She had been delivered of a child but a few months previous to her admiffion; and as her fituation in life could not be fuppofed to be fuch as to have allowed thofe neceffary indulgences which a peculiar and delicate conftitution, both before and after delivery, fometimes demands, I was inclined at firft rather to attribute her diforder to a morbid irritability of the ftomach, increafed beyond what it was originally by a general wafte and debility of the whole body. Accordingly fhe was ordered to begin with the effervefcing faline mixture, to which was added a fmall portion of the tincture of opium, and to repeat repeat it after proper ſtated intervals. Some port wine was alſo allowed her, of which ſhe was directed to take a ſmall quantity as often as her ſtomach would bear it, without inconvenience, in the courſe of the day.

On the 1ſt of September ſhe had a decoction of the bark, with a little of the tincture, to make trial of every five or ſix hours, and the day following, her vomiting not being in any wiſe abated, but returning as uſual, the conſtituent parts of the ſaline draught were adminiſtered ſeparately, that the decompoſition of the mild vegetable alkali might take place within the ſtomach.

It ſeems unneceſſary to detail any farther the few other medicines employed on this occaſion: ſuffice it to ſay, that all were equally ineffectual in preventing the return of thoſe acts of vomiting to which ſhe had been ſo long unhappily accuſtomed: I began, therefore, ſoon to miſtruſt whether I was right in aſcribing thoſe repeated rejections of the ſtomach ſolely to an increaſed irritability of that organ; and from a more minute inquiry into paſt circumſtances, and a ſtricter attention to the preſent, it appeared to me ſufficiently manifeſt that the real cauſe of her diſorder conſiſted in a ſcirrho-

ſity fity of the parts furrounding the pylorus, in confequence of which the food in the ftomach was prevented from paffing fo readily into the duodenum as it ought. Her vomiting generally came on about three or four hours after the taking of food. She was coftive, and complained of fome pain at the region of the pylorus upon its being preffed; but the hardnefs which, in fimilar cafes, is commonly felt about that part was, however, but imperfect and indiftinct.

Although thoroughly perfuaded as to the nature of the complaint, yet I was unwilling, from the great degree of debility and emaciation to which fhe was reduced, to order any other medicines but fuch from time to time as were fitted merely to alleviate the different fupervening fymptoms, with a view to render her few remaining days as comfortable and eafy as poffible. The cicuta and calomel, which, if they had been given at the commencement of the diforder, might poffibly either have effected its removal, or retarded its progrefs, I omitted to make trial of, being of opinion that no permanent relief could be derived from the ufe of them after the obftruction had been increafing fo long, and had occafioned fo great a degree of

 weakweaknefs : I waited, therefore, with a full affurance that death would fhortly put a period to all her miferies, which accordingly happened on the 10th of October following, the powers of life having been fairly exhaufted for want of their ufual affiftance and fupport.

On laying the contents of the abdomen into view, the ftomach prefented itfelf uncommonly enlarged, with about three pints of a thick whitifh matter contained in it, which feemed chiefly to confift of bread and milk, which conftituted the principal part of her food fome days before her death. The whole circular border, which forms the pylorus, leading from the ftomach into the duodenum, was found to be quite hard and fcirrhous, and the pylorus itfelf was found fo much diminifhed in fize as to be nearly obliterated.

There are feveral cafes upon record of a fcirrhous pylorus. Sauvages, in his *Nofologia methodica*, makes mention of three which fell under his own immediate obfervation. In the feveral works of Hoffman *, Willis †, and

* Opera omnia phyfico-medica, Genev. edit. 1748. Tom. II. p. 260.

† Opera omnia, Tom. II. p. 24.

Bonetus,

Bonetus *, ſome inſtances of this nature are to be found. Kerckringius † relates a very ſingular caſe, in which a piece of money that had been ſwallowed occaſioned a fatal termination by forming an obſtruction at the pylorus. The ſcirrhus in ſuch caſes is ſeldom confined ſolely to thoſe parts which immediately ſurround the pylorus. Sometimes it ſeizes the upper extremity of the duodenum, and ſometimes alſo it ſeizes other neighbouring parts. The beſt manner of diſcovering any calloſity or hardneſs in theſe parts is to examine them minutely while the patient is lying down, with the knees bent and raiſed.

It is eaſy to perceive the reaſon why the vomiting in theſe caſes ſhould occur ſo regularly a few hours after the taking of nouriſhment. The food, in conſequence of not being timely propelled into the inteſtines, but remaining in the ſtomach, ſoon acquires an irritating quality ſufficient to affect this tender organ, ſo as to throw it into action, whereby its contents are either wholly or partially diſcharged.

* Sepulcretum, ſive anatomia practica, de Vomitu, Obſ. 17, & aliæ.

† Obſerv. anatom. 1.

It muſt be a painful and mortifying circumſtance to any one to ſee a diſorder, like the foregoing, bringing on a ſlow and gradual deſtruction of the human conſtitution, without being able to afford any ſort of aſſiſtance either towards occaſioning its removal or retarding its progreſs; yet when the cauſe is detected by the medical practitioner, and known to be ſuch as to be altogether immoveable, he enjoys, as to the fatal iſſue of the complaint, a degree of ſatisfaction which can accompany thoſe only who are juſtly entitled to it. It is, however, of the higheſt importance in complaints ariſing from the above mentioned cauſe, as well as in moſt others, to aſcertain with preciſion, at an early period, the true nature of the diſeaſe. If medicine is ever likely to miniſter its aid, no period of the diſorder can give it a greater probability of ſucceeding than the firſt; and in caſes where the general health is not yet materially injured, there remains much reaſon to hope that from a ſuitable courſe of remedies, duly perſevered in, a removal of the obſtruction might at length be accompliſhed.

*Sherborne,*
Sept. 25, 1790.

V. *Some*

